# Strengthening healthy aging through community-based integrated care: A study protocol evaluating functional decline and quality of life among middle-aged and elderly tribals in Jharkhand

**DOI:** 10.1371/journal.pone.0358356

**Published:** 2026-09-21

**Authors:** Kantika Yadav, Syed Irfan Ali, Jarina Begum, Swati Shikha, Abhishek Kumar, Pallavi Lohani

**Affiliations:** Department of Community Medicine, Manipal Tata Medical College, Manipal Academy of Higher Education, Manipal, India; The Foundation for Medical Research, INDIA

## Abstract

**Background:**

India is undergoing a significant demographic shift, with the elderly population (60+) projected to reach 20% by 2050. Tribal populations, such as those in Jharkhand, face accumulated disparities, including premature aging, lower life expectancy (63.9 years), and a dual burden of communicable and non-communicable diseases. Despite these challenges, there is a substantial research gap regarding early-onset functional decline—often beginning at age 45 due to physical labour and undernutrition—and the lack of culturally adapted healthcare models for these underserved groups.

**Objectives:**

This study aims – Phase 1 (Needs Assessment): Assess functional decline, quality of life (QoL), and prevalent health conditions/risk factors among middle-aged and elderly tribal adults in East Singhbhum, Jharkhand and Phase 2 (Model Development): Design and evaluate the feasibility and acceptability of a culturally sensitive, community-based integrated healthcare model to improve functional ability and QoL.

**Methods:**

The study employs a two-phase concurrent mixed-methods study design. Setting & Participants: Conducted in 25 villages across five tribal-dominated 5 blocks of East Singhbhum, targeting 520 tribal individuals aged 45 years and above. In Phase 1, Quantitative Component: A cross-sectional needs assessment will use tools like Barthel’s Activity of Daily Living (ADL), Lawton’s Instrumental Activities of Daily Living (IADL), and WHOQOL-BREF to establish baseline health and functional data. Qualitative Component: In-depth interviews, focus group discussions, and key informant interviews with community members, traditional healers, and health workers will explore contextual barriers and cultural perceptions of aging. Quantitative and qualitative findings will be analysed independently and integrated to identify priority domains for healthcare model development. In Phase 2, these findings will inform the co-development of a culturally sensitive, community-based integrated healthcare model through stakeholder consultation. The model will subsequently be implemented in a selected village to assess its effectiveness, feasibility, and community acceptability before refinement.

**Expected Outcomes:**

The study will establish critical baseline data on tribal aging and develop a validated, scalable healthcare model. Expected results include improved health service utilization, early detection of functional decline, and policy-level impact on geriatric and tribal health strategies in India.

**Conclusion:**

By prioritizing vulnerable populations and integrating indigenous knowledge with formal health systems, this protocol aligns with Sustainable Development Goals (SDGs 3, 10, and 17) to promote social equity and lifelong well-being for India’s tribal communities.

## Introduction

India is undergoing a major demographic and epidemiological transformation, with an increasing proportion of its people reaching middle and old age. The population aged 60 and above is predicted to increase considerably, rising from 8.6% in 2011 to nearly 20% by 2050, indicating a significant demographic shift towards an older population [1]. This trend is accompanied by an increase in chronic noncommunicable diseases, functional limits, and social vulnerabilities, which disproportionately affect underprivileged people, such as Scheduled Tribes. The ageing trajectory and health burdens of tribal groups, particularly in resource-constrained and physically isolated locations such as Jharkhand, have yet to be thoroughly investigated via empirical research [2].

Geographic, socio-cultural, and economic constraints frequently result in accumulated disparities within tribal communities. These variables can contribute to premature ageing, undiagnosed chronic diseases, and low quality of life, necessitating early detection and care [3]. Functional decline, defined as a decreased capacity to execute basic and Instrumental activities of daily living IADLs), is a strong predictor of disability, healthcare demands, and reduced independence in later life [4]. Quality of life, which is determined by physical, emotional, social, and environmental aspects, is also important in comprehending the whole experience of ageing [5].

The tribal population in India has much lower wellness scores than the mainstream population, the projected life expectancy from birth is 63.9 years versus 67 years countrywide [6]. Communicable diseases disproportionately afflict tribal people, accounting for 80% of malaria cases and 50% of associated deaths. The disease burden of pulmonary tuberculosis in tribal groups is substantially higher, at 703 per 100,000, compared to 256 per 100,000 in the general population. Noncommunicable diseases such as hypertension, diabetes, cardiovascular disease, and cancer are increasingly being reported in tribal areas. Particularly Vulnerable Tribal Groups (PVTGs) have high rates of malnutrition and genetic diseases such as sickle cell anaemia and G6PD deficiency [7].

In India, older adults are predominantly located in rural areas., which have a significantly greater prevalence of functional disability (34.3%) than urban areas (29.9%). Despite this gap, research has primarily concentrated on nutritional status and morbidity, with little emphasis on functional status. There is still a major vacuum in studies that especially address functional disability among the rural elderly [4].

Considering these developments, the current study seeks to assess functional decline, identify prevalent health disorders and risk factors, and evaluate quality of life among middle-aged and elderly tribal adults in selected districts of Jharkhand. The findings will provide the basis for developing culturally relevant, community-based care models in tribal settings that promote healthy ageing and prevent long-term dependency.

## Literature review

Tribal groups in India have unique health and social challenges, particularly as they age. A growing body of literature has identified these disparities and gaps in health service delivery, which this study aims to address through a community-based integrated care approach tailored to indigenous people.

To achieve universal health coverage, Reddy K.S. suggested significant changes to India’s healthcare system. They emphasized prioritizing vulnerable groups and investing in grassroots health systems, which is consistent with the study’s goals of delivering equitable care to senior tribal members.

Soren studied the impact of social isolation, comorbidities, and economic dependency on the Quality of life of old people in a tribally dominant state. This emphasizes the importance of multidimensional care, as promoted by this paradigm [3].

Kumar assessed frailty among old tribals, revealing early functional decline caused by poor diet and rigorous labor. The findings bring attention to the necessity of implementing preventive approaches and regular health assessments in vulnerable communities, which this approach aims to overcome [1].

Roy evaluated the issues faced by India’s tribal health system and compared them to global healthcare norms. Their research focuses on systemic concerns such as inadequate healthcare infrastructure, cultural hurdles, and poor healthcare access greatly affects tribal groups.. The authors underline the significance of culturally relevant healthcare interventions, as well as major investments in tribal health infrastructure, to close these gaps. Their findings are consistent with the goals of this study, which aims to address these disparities for older Indigenous populations using a culturally relevant, community-based integrated care strategy [2].

Negi highlighted the double burden faced by tribal communities in India due to historical marginalization and inadequate access to healthcare services. Despite their rich heritage of traditional healing practices, tribes are increasingly experiencing a loss of cultural health knowledge alongside limited outreach of modern medical systems. The authors emphasize that tribal populations are disproportionately affected by both communicable and non-communicable diseases, compounded by poor infrastructure, low literacy, and socio-economic deprivation. The study argues for the integration of traditional health systems with modern public health approaches to ensure culturally appropriate and effective care. It concludes with a call for context-sensitive policies and inclusive health strategies tailored to the unique needs of India’s tribal populations [3].

The Longitudinal Ageing Study in India (LASI), launched in 2017–18 by the Ministry of Health and Family Welfare in collaboration with IIPS, Harvard University, and USC, stands as the first nationally representative survey to comprehensively assess health, functional status, and quality of life among individuals aged 45 and above. While LASI provides critical insights into the ageing process in India, there remains a significant data gap concerning the early onset of functional decline and associated determinants among middle-aged and elderly tribal populations, especially the Particularly Vulnerable Tribal Groups (PVTGs) [5].

## Research gap identified

Inadequate Customization of Health Policies for Tribals (Reddy et al., 2020): Research in this area is currently sparse on how universal health policies (like UHC) can be adapted to suit the distinct sociocultural and infrastructural realities of tribal populations, particularly the elderly.

Neglect of Psychosocial Determinants of Ageing (Soren et al., 2022): Elderly tribals’ quality of life is insufficiently studied, especially factors like social isolation, economic dependence, and erosion of traditional community support systems.

Limited Understanding of Functional Decline and Frailty (Kumar et al., 2023): Research on frailty, nutritional status, and the impact of lifelong physical labor on functional capacity in tribal elders is sparse, leading to poor early identification and intervention.

Weak Integration of Traditional and Modern Healthcare Systems (Deb Roy et al., 2023): There’s a clear gap in how indigenous tribal health practices can be scientifically studied and effectively integrated with formal health systems without compromising cultural identity.

Lack of Context-Specific, Empirical Tribal Health Research (Emerald, 2021): Existing studies do not adequately capture the structural barriers, cultural nuances, and geographical challenges that shape health-seeking behavior and outcomes in tribal regions. Empirical, on-ground evidence is missing.

## Objectives

### Primary objective

#### Phase 1-Needs assessment.

1. To assess functional decline and determine prevalent health conditions and risk factors among middle-aged and elderly tribals, in selected blocks of East Singhbhum district.
2. To evaluate the quality of life and associated health determinants, including prevalent risk factors, among the same population groups in the study area.

### Secondary objective

#### Phase 2- Healthcare model development.

3. To design a community-based, culturally sensitive healthcare model to assess its feasibility and acceptability in improving functional ability and Quality of life across middle-aged and elderly tribal populations across all the blocks of East Singhbhum.

## Detailed methodology

### Overall research design

**Fig 1:**
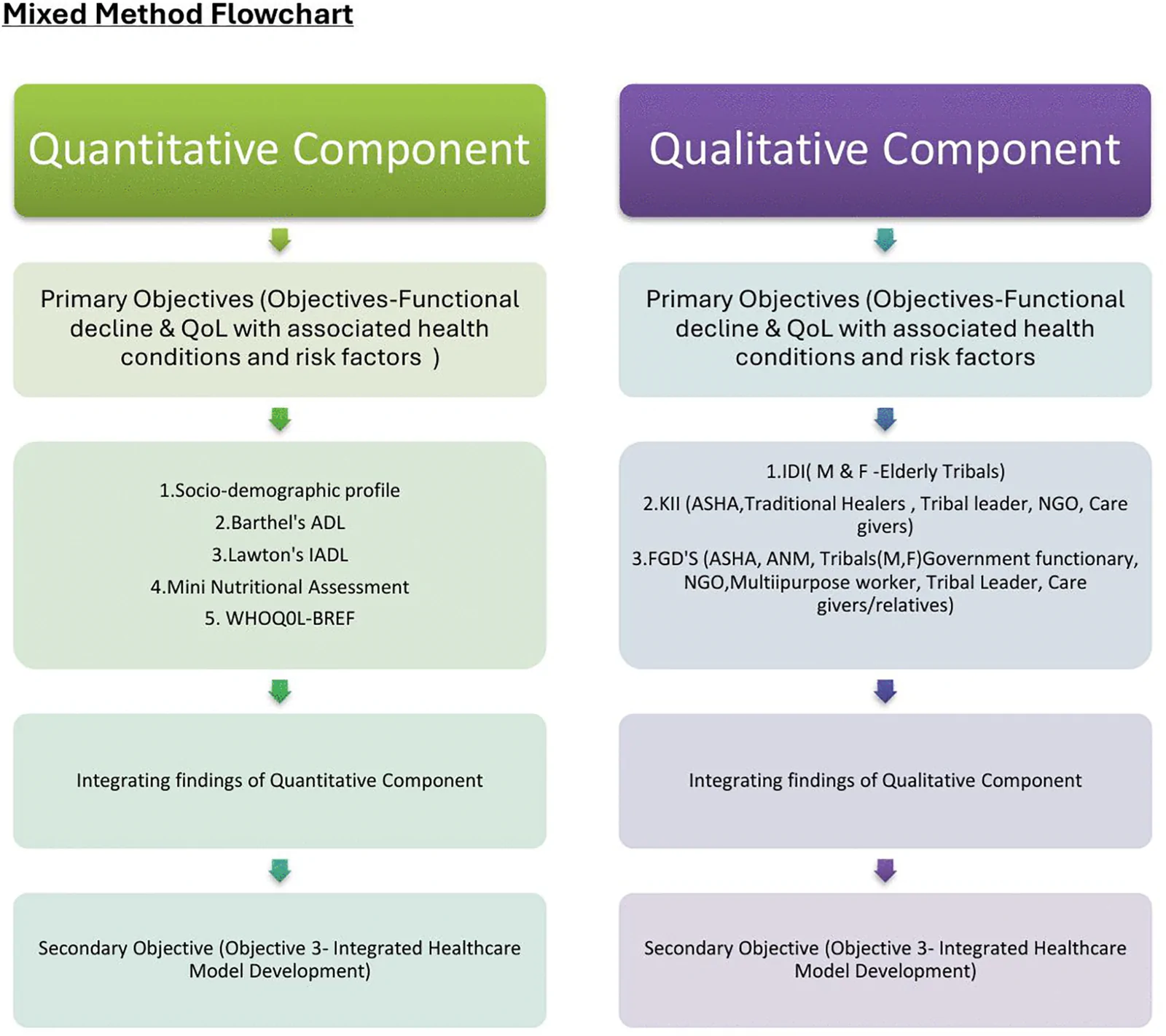
Overview of the concurrent mixed-methods study design integrating quantitative and qualitative components for assessing functional decline and quality of life, informing the development of a community-based integrated healthcare model.

The study will employ a two-phase mixed-methods implementation research design. Phase 1 will adopt a concurrent mixed-methods approach, in which quantitative and qualitative data will be collected simultaneously to comprehensively assess functional decline, quality of life, health conditions, healthcare utilisation, and socio-cultural determinants among middle-aged and elderly tribal populations in Jharkhand. The quantitative and qualitative findings will be analysed independently and integrated during interpretation to provide a comprehensive understanding of the community’s health needs. The integrated findings from Phase 1 will be used to identify, prioritise, and confirm the key domains that will underpin the development of the healthcare model in Phase 2 Fig 1. Building on these empirically derived domains, Phase 2 will focus on the co-development of a culturally sensitive, community-based integrated healthcare model through evidence synthesis and stakeholder consultation. This iterative process ensures that the proposed model is evidence-based, context-specific, and responsive to the health priorities, cultural practices, and service needs of tribal [8].

### Trial registration

The study has been prospectively registered with the Clinical Trials Registry–India (CTRI) (Registration No. CTRI/2026/04/108631).

### Study setting

The study will be conducted in East Singhbhum district, Jharkhand, India, a tribal-dominated district with substantial populations of Scheduled Tribes (STs) and Particularly Vulnerable Tribal Groups (PVTGs). Data collection will be undertaken across five tribal-dominated blocks—Golmuri–Jugsalai, Potka, Ghatshila, Dhalbhumgarh, and Ghurabandha—to capture the district’s geographical, socio-cultural, and healthcare diversity. Using a multistage sampling approach, 25 villages (five villages per block) will be selected to ensure comprehensive within-district representation and to generate evidence for the development of a culturally sensitive, community-based integrated healthcare model for middle-aged and elderly tribal populations.

### Study population

The study population will comprise tribal individuals aged 45 years and above residing in rural and tribal-dominated regions of East Singhbhum district, Jharkhand, including both men and women belonging to Scheduled Tribes (STs) and Particularly Vulnerable Tribal Groups (PVTGs). Individuals aged 45 years and above have been selected because tribal populations in India experience premature ageing due to chronic undernutrition, physically demanding occupations, poverty, environmental hardships, and limited access to healthcare services. Consequently, functional decline, multimorbidity, and other age-related conditions often occur earlier than in the general population. Restricting the study to individuals aged 60 years and above may therefore overlook a substantial proportion of tribal adults already experiencing early functional impairment and poor quality of life. Furthermore, the relatively lower life expectancy among tribal populations (approximately 62–65 years) supports the inclusion of middle-aged adults to enable earlier identification of health needs and generate evidence for the development of a culturally sensitive, community-based integrated healthcare model.

### Eligibility criteria

Inclusion

- The participant must be 45 years of age or older.
- The individual should belong to a Scheduled Tribe (ST) or a Particularly Vulnerable Tribal Group (PVTG).
- The participant must have been a continuous resident of the selected area for at least five years.
- The participant must participate voluntarily in the study and able to provide informed consent

Exclusion

- Individuals who have severe cognitive impairment that prevents them from understanding or responding to interview questions will be excluded from the study.
- Participants who are currently enrolled in similar health-related interventions or research studies will not be included, to avoid duplication and potential bias in the findings.

### Sample size

#### Quantitative component.

The sample size was determined using Cochran’s formula for large populations, utilizing the reported 34.3% prevalence of functional disability among rural older adults from the Longitudinal Ageing Study in India (LASI) Wave 1 as the reference estimate [5].

This reference proportion (p) was applied alongside a 95% confidence level and a 5% margin of error to calculate an initial requirement of 346 participants. To account for the multistage cluster sampling design, a design effect of 1.5 was applied, resulting in a final target sample size of 520. The formula used here is as follows:


$$\begin{array}{c} n\;=\frac{\left(z^{\text{2}}*\;\hat{p}\left(\text{1}\;-\;\hat{p}\right)\right)}{\varepsilon^{\text{2}}}\;(\text{4}) \end{array}$$

where:

- n = required sample size
- Z = 1.96 (95% confidence level)
- p = 0.343 (estimated prevalence of functional disability)
- 1 − p = 0.657
- d = 0.05 (absolute precision)

Substituting these values:

n=(0.05)2(1.96)2×0.343×0.657=346

As the study will employ a multistage cluster sampling approach, a design effect (DEFF) of 1.5 was applied to account for clustering of observations. This yielded a final sample size of:

346 × 1.5 = 519

which was rounded to **520 participants.**

#### Qualitative component.

The qualitative component will employ convenient sampling and include 10 In-Depth Interviews (IDIs) (one male and one female participant from each of the five study blocks), 5 Focus Group Discussions (FGDs) (one per block), and 12 Key Informant Interviews (KIIs) involving ASHAs, Mukhiyas, NGO representatives, traditional healers, Medical Officers, and other key stakeholders. The proposed methods and sample distribution are presented in Table 1. The proposed sample size is based on the principle of thematic saturation and is expected to provide adequate representation across gender, age, and stakeholder groups. Data collection will continue until thematic saturation is achieved; however, if new themes emerge, additional interviews (up to 20% of the proposed sample) may be conducted after notification to the Institutional Ethics Committee (IEC). Data collection will continue until thematic saturation is reached.

**Table 1:** Qualitative data collection methods and sample distribution.

Method	Convenient Sampling	Total
IDIs	2(Male, Female from each block)	10
FGDs	1 per block	5
KIIs	(ASHA, Mukhiya, NGO’S, local leader, Traditional Healers, Medical Officers)	12

### Sampling strategy

#### Quantitative component.

**Fig 2:**
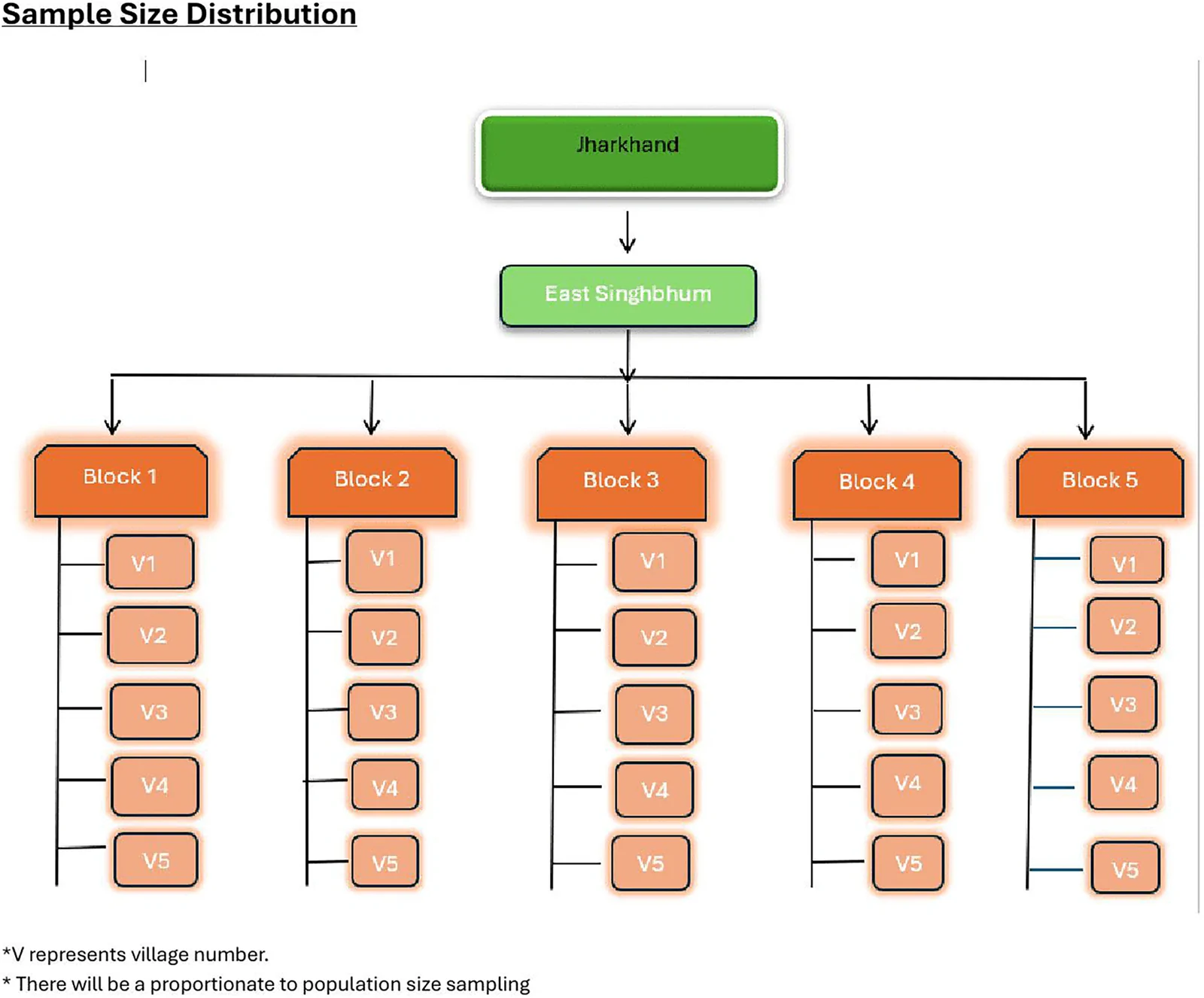
Representing sample size distribution in 5 blocks and 25 villages respectively.

A multistage stratified random sampling strategy will be employed for the quantitative component. In the first stage, five tribal-dominated blocks of East Singhbhum district (Golmuri–Jugsalai, Potka, Ghatshila, Dhalbhumgarh, and Ghurabandha) will be included. In the second stage, five villages will be randomly selected from each block, resulting in a total of 25 study villages. The distribution of the sample across the five blocks and 25 selected villages is illustrated in Fig 2. Finally, eligible participants aged ≥45 years will be selected using proportionate allocation and systematic random sampling within each selected village until the required sample size is achieved Table 2–5.

**Table 2:** Depicting Operational Framework for Replication of Healthcare.This model ensures sustainability by fostering community ownership through tribal leaders, village councils and tribal volunteers, moving beyond disease-centric care to a holistic approach that tracks autonomy and quality of life.

S.No.	Intervention Pillar	Key Strategies	Replicability Features
1.	Outreach	Monthly mobile clinics & AYUSH integrated geriatric-NCD screening.	SOP-driven screening; fixed calendar communicated via Sahiyas.
2.	Digital	Elderly Day with teleconsultations for remote access to specialists.	Digital attendance logs; CHO-managed video/audio sessions.
3.	Entitlement	Convergent enrollment in Ayushman Bharat & pension schemes.	Household entitlement checklists; inter-sectoral coordination.
4.	Capacity	ToT Model for Sahiyas, Healthcare workers and community volunteers in functional care.	Cascade training protocols; 6-month refresher training.
5.	Traditional	Validation of tribal healing practices by AYUSH doctors.	Standardized safety & validation checklists for healers.
6.	Nutrition	Training on MNA tools, kitchen gardens, and tribal diet promotion.	Alignment with Poshan Abhiyan; local agricultural synergy.
7.	Psychosocial	Block-level support groups & home-based physical/mental/social rehab.	Facilitator guides for SHG-linked elderly support groups.

**Table 3:** Core components of the proposed community-based integrated healthcare model.

Category	Key Details & Strategies	Unique Features / Strategic Role
**Contextual Assessment**	**Strengths:** Close-knit community networks, reliance on traditional healing, and indigenous knowledge systems. **Weaknesses:** Limited formal infrastructure, staff shortages, low literacy, and economic vulnerability.	**Strategic Role:** These insights guide the tailoring of interventions, ensuring the model builds on community strengths while addressing critical gaps.
**Approach to Healthcare**	**Community-Based:** Delivered through health workers, mobile units, and local organizations. **Orientation:** Preventive and promotive (early screening, health education, lifestyle modification).	**Holistic Approach:** Focuses on combining physical, mental, and social well-being rather than exclusively focusing on disease management.
**Healthcare Delivery System**	**Primary:** ASHAs, ANMs, and volunteers for screening and home visits. **Secondary:** CHCs/District Hospitals with dedicated geriatric/NCD clinics. **Tertiary:** Referral links to medical colleges.	Establishes a **structured referral and monitoring pathway** customized for the tribal landscape.
**Integration with Existing Programs**	Leverages **NPHCE, NPCDCS, NHM**, and national nutrition programs such as **ICDS and Poshan Abhiyan**.	**Unique Feature:** Provides programmatic convergence customized for tribal/elderly needs, which is rarely done systematically in existing models.
**Traditional Healing Practices**	Recognizes and documents traditional healers; promotes safe, evidence-based practices while discouraging harmful ones.	**Unique Feature:** Structured collaborative engagement with traditional healers to improve the community’s trust and acceptability of modern healthcare.
**Nutritional Practices**	Incorporates **tribal diets** and local food diversity; introduces low-cost, culturally acceptable interventions like kitchen gardens.	**Unique Feature:** Blends nutrition security directly with healthcare delivery rather than treating them as separate entities.
**Elderly-Focused Interventions**	Builds on NPHCE but is adapted for tribal settings, including **functional decline (ADL/IADL)** and quality of life assessments	**Unique Feature:** Adds community-based care models that specifically account for caregiver burden, social isolation, and financial dependence.

**Table 4:** Representing outcome measures and assessment timeline.

S.No.	Timeline	Outcome Components	Measurement Tools & Methods
1.	Phase1: Needs Assessment (Completion by November 2026	Baseline Functional Status, QoL, and Morbidity	• Barthel’s Index (for ADL) and Lawton’s Scale (for IADL). • WHOQOL-BREF questionnaire for Quality of Life. • Long MNA (Mini Nutritional Assessment) for nutritional status.
2.	Phase1: Qualitative Inquiry (Concurrent with quantitative)	Cultural Beliefs and Stakeholder Engagement	• In-Depth Interviews (IDIs), Focus Group Discussions (FGDs), and Key Informant Interviews (KIIs) with leaders, healers, and health workers.
3.	Phase 2: Model Implementation (June – August 2027)	Short-term Model Effectiveness	• Pre-post measures of functional ability and quality of life using the Phase 1 quantitative tools.
4.	Phase2: Evaluation (Sept – Nov 2027)	Feasibility, Acceptability, and Service Utilisation	• Qualitative feedback from participants and stakeholders to refine the model. • Tracking utilisation rates and health screening participation.
5.	Long-Term (18 + months and beyond)	Sustained Improvement and Policy Impact	• Monitoring reduction in functional decline rates and chronic disease complications. • Documenting state-level institutional adoption and policy inputs

**Table 5:** Data Management Plan outlining data ownership, collection, storage, anonymisation, retention and ethical compliance procedures.

S.NO.	SECTION	DETAILS
1.	Data Ownership	All data generated from this study will be considered the intellectual property of the Department of Community Medicine.
2.	Data Collection	• Data will be collected through questionnaires, informed consent forms, in-depth interviews, focus group discussions, and key informant interviews.• Audio and/or video recordings (where consented) will be transcribed and anonymized.
3.	Data Storage & Access	• Hard Copies:- Questionnaires, consent forms, and field notes will be stored in a locked almirah within the Department of Community Medicine.- The key will remain with the research scholar.• Electronic Data:- Digital files (including transcripts, recordings, and datasets) will be password-protected.- Access will be restricted to the Principal Investigator and the Research Scholar.- Files will be stored on secure institutional computers, with no use of personal devices or unsecured cloud storage.
4.	Data Anonymization	• All participant identifiers will be removed during transcription and data entry.• Pseudonyms or unique ID codes will be used instead of real names.• Publications and reports will not disclose any identifying information.
5.	Data Retention and Disposal	• Data will be maintained until the completion of the PhD work and subsequent publication.• After completion:- Electronic data will be archived securely or deleted as per institutional policy.- Hard copies will be shredded/incinerated or archived according to institutional guidelines.
6.	Ethical Compliance	• The study will follow Institutional Ethics Committee (IEC) guidelines for data confidentiality and participant rights.• Any breach or concern regarding data security will be immediately reported to the Department and IEC.

#### Qualitative component.

The qualitative component will employ convenient sampling to recruit participants with diverse demographic and experiential characteristics. Participants will include middle-aged and elderly tribal community members, frontline health workers, traditional healers, community leaders, Medical Officers, and representatives of local organizations to ensure a broad range of perspectives relevant to healthy ageing and healthcare delivery from five tribal-dominated blocks of East Singhbhum district. The proposed methods and sample distribution for the qualitative component are presented in Table 1.

### Study procedure

#### Phase 1: Concurrent mixed-methods needs assessment.

Phase 1 will focus on conducting a comprehensive needs assessment among middle-aged and elderly tribal populations, in the 5 blocks of East Singhbhum with better operational feasibility.

#### Phase 1 (needs assessment).

Objectives

1. To assess functional decline and determine prevalent health conditions and risk factors among middle-aged and elderly tribals, in selected blocks of East Singhbhum district.
2. To evaluate the quality of life and associated health determinants, including prevalent risk factors, among the same population groups in the study area.

The quantitative component will employ a community-based cross-sectional design to assess functional status, quality of life, morbidity, nutritional status, and associated risk factors among tribal individuals aged 45 years and above. Following written informed consent, trained investigators will administer a structured interviewer-based questionnaire during household visits. Standardized and validated instruments, including the Barthel Activities of Daily Living (ADL) Index [7], Lawton Instrumental Activities of Daily Living (IADL) Scale [9], WHOQOL-BREF [10] and relevant clinical assessments, will be used to evaluate functional ability, quality of life, nutritional status, and health conditions. Data will be collected from approximately 520 participants selected through a multistage stratified random sampling approach across 25 villages in five tribal-dominated blocks of East Singhbhum district. The findings will identify health needs and priority domains to inform the development of the community-based integrated healthcare model in Phase 2.

The qualitative component will provide contextual and experiential insights into functional decline, healthy ageing, and healthcare needs among tribal populations.

The study will use convenience sampling to capture diverse perspectives across age groups, gender, and social roles.

- Participant Groups: Middle-aged (45–59 years) and elderly (≥60 years) tribal individuals, including members of Scheduled Tribes (STs) and Particularly Vulnerable Tribal Groups (PVTGs).
- Key Informants: KIIs will be conducted with frontline health workers (Sahiyas/ASHAs, ANMs, AWWs), traditional tribal healers, local leaders (Mukhiyas), and Medical Officers to explore cultural practices, healthcare access, and health-system perspectives.
- FGD Stratification: FGDs will be stratified by age group (45–59 and ≥60 years) and, where feasible, by gender to explore differences in health-seeking behaviour and caregiving experiences.
- Planned Sample: The qualitative component will include 10 IDIs, 5 FGDs (one per block), and 12 KIIs, with final sample size guided by thematic saturation.

#### Phase 2: Development of the community -based healthcare model.

Objective 3. To design a community-based, culturally sensitive healthcare model to assess its feasibility and acceptability in improving functional ability and Quality of life across middle-aged and elderly tribal populations across all the blocks of East Singhbhum.

**Fig 3:**
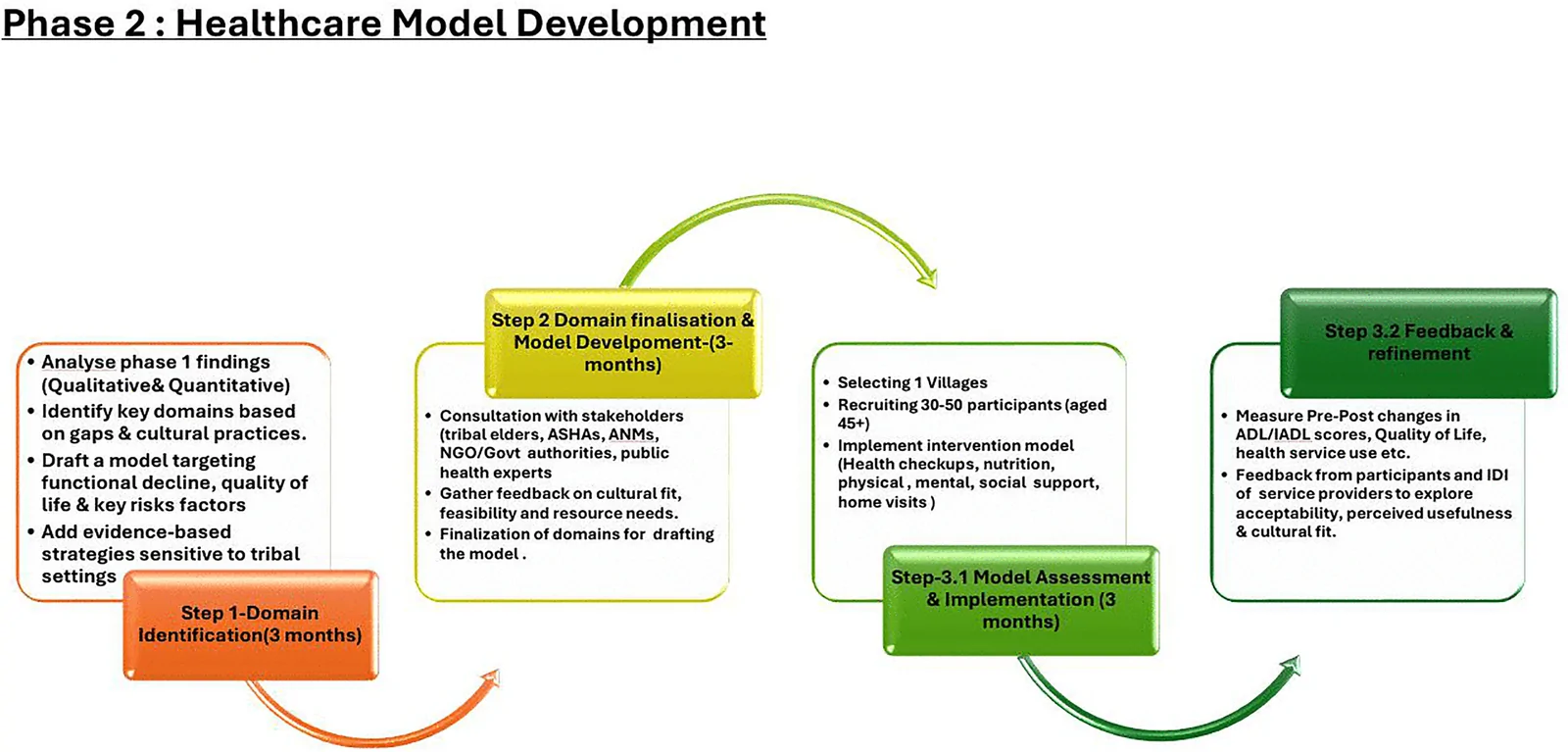
Phased framework for development and implementation of a community-based integrated healthcare model by assessing the feasibility and acceptability for improving functional ability and quality of life among the tribal population in East Singhbhum.

Phase 2 will build on the integrated quantitative and qualitative findings from Phase 1 to fulfil Objective 3, which involves developing a culturally appropriate, community-based integrated healthcare model for healthy aging among tribal populations. The identified domains from Phase 1 will be refined through stakeholder consultations involving tribal community representatives, Medical Officers, traditional healers, local leaders, public health experts, and other relevant stakeholders. Support from ASHA/Sahiya workers and local community stakeholders will be sought throughout participant mobilisation, community engagement, and implementation of the healthcare model Fig 3. Based on the consensus generated, the healthcare model will be developed and implemented in a selected village to assess its effectiveness, feasibility, and community acceptability. Written informed consent will be obtained from all participants prior to their involvement, either through signature or thumb impression.

The development of the healthcare model will follow a systematic approach beginning with an in-depth analysis of phase 1 findings. It will be conducted in the following three steps.

#### Step 1: Identification and Confirmation of Healthcare Model Domains.

Phase 1 findings (both quantitative and qualitative) will be integrated and analysed to identify priority key health needs, functional decline patterns, healthcare service gaps, and socio-cultural determinants. Evidence from the literature and existing healthy ageing frameworks will further support the identification and confirmation of the core healthcare model domains.

#### Step 2: Stakeholder Consultation and Healthcare Model Development.

A draft model will be developed using evidence-based, culturally appropriate strategies. It will be refined through stakeholder consultations (tribal leaders, ASHAs, NGOs, public health experts, Pradhan etc.) to ensure feasibility and acceptability, leading to finalisation of domains and model. A gender-stratified approach will guide tailored interventions. The healthcare model will adopt a gender-stratified approach, using questionnaire findings to identify gender-specific health needs, risks, and barriers and design tailored interventions. For men, this will focus on non-communicable diseases, substance abuse, and occupational risks, while for women, it will address reproductive health, nutrition, chronic conditions, and caregiver burden. Service delivery will also be adapted, with community or workplace outreach for men and mobile camps, home visits, and empowerment initiatives for women, ensuring a holistic response to biological, social, and cultural determinants of health.

**Step 3: Implementation.**

### Assessment of the effectiveness of the model

The finalised model will then be implemented in a selected village, incorporating components such as health checkup, nutrition support, physical and mental health interventions, and home-based care. Further the effectiveness of the model will be evaluated using pre-post measures of functional ability and quality of life.

### Feedback and refinement

Qualitative feedback from participants and key stakeholders will assess feasibility & acceptability. Their perspectives will be systematically elicited, analysed, and incorporated into the model’s refinement.

### Detailed intervention outline

#### HOR-SATHI Healthy Ageing Model.

**Component A: H: Health Outreach & Clinical Care**

1. Monthly Mobile Healthcare Clinics to be staffed by Community Health Officers (CHOs) or ANMs, with rotating support from Medical Officers.
2. Regular WHO ICOPE-based geriatric-NCD screening for cognitive, locomotor, nutritional, psychological, visual and auditory impairments, along with blood pressure, blood glucose, and functional status assessment, followed by risk-based referral and care.
3. Integration of AYUSH services by having an AYUSH doctor or qualified practitioner on hand in mobile clinics to offer supportive management for chronic diseases based on Ayurveda or yoga in addition to traditional allopathic treatment.

**Component B: O: Optimal Nutrition & Well-being**

1. Training Sahiyas and Anganwadi workers in nutritional assessment, including the use of the Mini Nutritional Assessment, and providing appropriate dietary counselling.
2. Promoting dietary diversity and kitchen gardens to increase the consumption of locally available, iron- and calcium-rich tribal foods.

**Component C: R: Rehabilitative & Functional Support**

Elderly Support Groups: Establishment of gender-stratified peer support groups to reduce social isolation and strengthen existing community networks through Self-Help Groups (SHGs)

- Multidimensional Rehabilitation
  - Mental: Counselling provided by trained personnel from the District Mental Health Programme.
  - Physical: Home-based rehabilitation exercises taught to family caregivers by trained volunteers, yoga instructors, or physiotherapists.
  - Social: Promoting mutual support, trust and collective participation within the tribal community through “Gram Chaupal” for better health.

**Component D: S: Social Protection & Entitlement Linkage**

1. Facilitate enrolment in Ayushman Bharat, Arogya Mandir services, and relevant social security and pension schemes.
2. Aadhaar Facilitation by coordinating with local Panchayat members, elderly tribal leaders to address documentation gaps and other practical barriers to scheme enrolment.

**Component E: A: Access through Community & Digital Connectivity**

Each month, the Sahiyas and CHOs organise a village-level event called “Elderly Day” to offer regular health examinations, health education, and chances for social interaction and community involvement.

- CHOs will facilitate teleconsultations, connecting older adults with off-site specialists, including geriatricians, dietitians, and AYUSH practitioners, for remote assessment and management.

**Component F: T: Traditional Medicine Validation**

1. Systematic documentation of local tribal healing practices and their role in community healthcare.
2. Local healing practices will be assessed by experts in AYUSH (Ayurveda, Yoga, Naturopathy, Unani, Siddha & Homeopathy) with safe and appropriate practices incorporated into the care pathway, while potentially harmful practices will be addressed through culturally sensitive, non-confrontational community education.

**Component G: H: Human Resource Capacity Building (Training-of-Trainers Model)**

1. Training of trainers- Master trainers (geriatric/public health faculty) will train CHOs/ANMs, who will subsequently train Sahiyas and selected grassroots-level healthcare workers and community volunteers, preferably male, to facilitate culturally appropriate engagement while maintaining gender balance and avoiding gender bias in community participation and service delivery.
2. Empowering Hamlets: Training volunteers from within the tribal community to extend healthcare services to remote areas with limited access to regular frontline health workers.

**Component H: I: I**ntegrated Care Coordination: Coordinating referrals, follow-up, and continuity of care across community, primary, secondary, and tertiary healthcare services.

**Operational Framework Table for Proposed Healthcare intervention**

#### Rationale behind the development of a community-based, culturally sensitive healthcare model.

The proposed healthcare model incorporates several innovative features that distinguish it from existing community-based and healthy ageing models in tribal settings. These features are intended to enhance cultural relevance, programme integration, and responsiveness to the healthcare needs of middle-aged and elderly tribal populations.

1. Age group inclusion-Includes participants belonging to both the middle-aged and elderly categories
2. Gender- and Age-Sensitive – Interventions are differentiated for men and women and specifically adapted for the elderly.
3. Integration of Modern & Traditional Systems- Acknowledges traditional healing instead of sidelining it, to improve community trust.
4. Nutrition-Care Linkage – Direct incorporation of local dietary practices and nutrition-sensitive interventions.
5. Community Empowerment – Uses existing social capital (Self Help Groups (SHGs), village councils) to drive awareness and decision-making.
6. Programmatic Convergence → Brings together NPHCE, NPCDCS, NHM, and nutrition schemes under one framework in tribal areas.

Monitoring Beyond Disease Outcomes → Tracks functional decline, quality of life, autonomy, and cultural acceptability — indicators usually ignored in mainstream programs.

#### Adaptability, scale-up, and sustainability of the model.

The proposed healthcare model is designed to support replication, scale-up, and long-term sustainability by integrating community-based care with existing health systems and national programmes.

Application in Other Settings (Replicability)

- Phased Development Framework: The model follows a three-step process of domain identification, model finalization, and implementation, allowing local health needs and socio-cultural factors to be assessed before intervention delivery.
- Early Intervention: Inclusion of individuals aged 45 years and above enables early identification and management of functional decline, particularly in resource-constrained and socially disadvantaged populations.
- Contextual Adaptation: The model begins with an assessment of local strengths and challenges, allowing interventions to be adapted to the infrastructure, resources, and needs of different communities.
- Cultural Adaptation: Culturally relevant strategies, including local dietary practices and indigenous knowledge, can be modified according to the cultural context of other populations.

Scaling Up the Model

- Programmatic Convergence: The model integrates existing programmes, including NPHCE, NPCDCS, NHM, and nutrition programmes such as Poshan Abhiyan, through coordinated referral and monitoring pathways.
- Institutional Adoption: Findings may support the adoption of the model by district- or state-level health authorities and its integration into existing health programmes.
- Expansion to Other Settings: Evidence generated through the pilot implementation may inform adaptation and scale-up in other tribal districts and settings with similar healthcare disparities.
- Policy Integration: The findings may provide evidence to inform future policies and programmes in geriatric and tribal health.

Long-Term Sustainability

- Community Ownership: Engagement of tribal leaders, Panchayats, NGOs, and other community stakeholders can promote local ownership and sustained participation.
- Local Capacity Building: Strengthening the capacity of Sahiyas/ASHAs and tribal volunteers can support continued community-level screening, health promotion, and home-based follow-up.
- Reduction of Economic Burden: Preventive and community-based care may help reduce avoidable healthcare expenditure and the financial burden associated with chronic disease and functional disability.
- Integration of Traditional Practices: Appropriate engagement with traditional healers may strengthen community trust and support culturally acceptable healthcare delivery.
- Use of Existing Social Structures: Engagement of Self-Help Groups (SHGs), village councils, and other established community networks can support continued health awareness, participation, and decision-making.

### Data collection method

The study will employ a two-phase mixed-methods implementation research design. Phase 1 will adopt a concurrent mixed-methods approach, in which quantitative and qualitative data will be collected simultaneously to comprehensively assess functional decline, quality of life, health conditions, healthcare utilisation, and socio-cultural determinants among middle-aged and elderly tribal populations in Jharkhand. The quantitative and qualitative findings will be analysed independently and integrated during interpretation to provide a comprehensive understanding of the community’s health needs. The integrated findings from Phase 1 will be used to identify, prioritise, and confirm the key domains that will underpin the development of the healthcare model in Phase 2. Building on these empirically derived domains, Phase 2 will focus on the co-development of a culturally sensitive, community-based integrated healthcare model through evidence synthesis and stakeholder consultation. This iterative process ensures that the proposed model is evidence-based, context-specific, and responsive to the health priorities, cultural practices, and service needs of tribal [8].

#### Quantitative component.

For the quantitative survey, villages from 5 blocks in East Singhbhum, Jharkhand, will be selected using multi-stage stratified random sampling to ensure representativeness of the tribal population. Within each selected village, eligible participants will be selected using random or systematic sampling methods, depending on feasibility (e.g., household listing followed by every nth household). The target population will include both middle-aged (45–59 years) and elderly (60 years and above) individuals. Data will be collected using structured questionnaires covering socio-demographic details, chronic disease profile, functional & nutritional status (e.g., Barthel’s Index, Lawton’s IADL, Long MNA), and quality of life (WHOQOL-BREF), and relevant clinical assessments, will be used to ensure consistency and reliability of data collection.

#### Qualitative component.

For the qualitative inquiry, convenient sampling will be employed to ensure diversity and representation across different age groups, genders, and tribal sub-communities. In-depth Interviews (IDIs), Focus Group Discussions (FGDs), and Key Informant Interviews (KIIs) will be conducted. Key stakeholders will include:

- Community members across age categories (middle-aged and elderly).
- Gender-specific groups to capture differences in health-seeking behaviour and caregiving roles.
- Community leaders and traditional healers to understand cultural perspectives and indigenous practices related to aging and health.
- Frontline health workers (ASHA, ANM, AWW’s etc.) Government officials and healthcare providers to explore perspectives on service delivery, challenges, and integration with government programs like NPHCE.

The qualitative tools will explore lived experiences of aging, perceptions of functional decline, caregiving dynamics, health system access, and acceptance of community-based healthcare interventions.

### Integration of the findings

The integration of quantitative and qualitative findings is a key component of the concurrent mixed-methods design. Both data strands will be collected simultaneously, analysed independently, and integrated during interpretation to provide a comprehensive and culturally sensitive understanding of the health needs of tribal populations.

1. **Integration at the Interpretation Stage**

The two data strands will first be analysed independently. Quantitative findings will provide measurable estimates and patterns of functional decline, chronic conditions, and quality of life, while qualitative findings will provide contextual insights into the underlying cultural, social, and healthcare-related factors, including perceptions of ageing, healthcare access, and traditional health practices. Integration of the findings will enable comparison and triangulation across methods and support development of an evidence-informed, context-sensitive model.

1. **Domain Identification**

The integrated Phase 1 findings will inform the identification and confirmation of the key domains for the healthcare model. Quantitative patterns, such as areas of functional limitation, will be considered alongside qualitative findings on service gaps and socio-cultural factors. This combined evidence will help identify and prioritize the health needs and service gaps that the intervention should address within the tribal context of East Singhbhum.

1. **Evidence-Based Model Refinement**

The integrated findings will inform the development of the draft healthcare model, which will subsequently be refined through consultations with stakeholders (tribal elders, ASHAs, ANMs, NGO/Government authorities, public health experts). For example, gender-specific patterns identified quantitatively will be considered alongside qualitative insights into caregiving roles and health-seeking practices to inform the development of context-specific interventions. Similarly, findings on morbidity and healthcare needs will be considered alongside information on traditional healing practices to promote culturally appropriate integration of healthcare services.

Overall, quantitative findings will establish the magnitude and distribution of identified health needs, while qualitative findings will provide contextual understanding to guide how these needs can be addressed within the cultural and health-system context of tribal communities.

### Data collection procedure

The data collection for the mixed-method study will begin by obtaining necessary approvals from the Institutional Ethics Committee (IEC), Institutional Scientific Committee (ISC), and administrative permissions from the State Tribal Welfare Department, District Health Authorities, and Panchayat-level leaders in the selected districts. Community sensitization meetings will be held in each selected village to inform local leaders, ASHA workers, and residents about the study’s purpose and procedures, thereby building trust and encouraging participation. A household listing will be conducted to identify eligible individuals aged 45 years and above, including both middle-aged (45–59 years) and elderly (60 + years) participants. Informed consent will be obtained, and for illiterate participants, thumb impressions will be taken in the presence of a witness to ensure ethical participation.

### Expected outcome

#### Primary outcome.

1. Functional Decline: Assessment of loss of capacity to perform basic and instrumental activities of daily living among middle-aged and elderly tribal individuals.
2. Quality of Life (QoL): Assessment of physical, psychological, social, and environmental well-being.
3. Health and Morbidity Profile: Assessment of prevalent chronic conditions, such as hypertension and diabetes, and associated risk factors at baseline.
4. Model Effectiveness: Assessment of short-term changes in mobility, ADL performance, self-rated health, and QoL following pilot implementation.

#### Secondary outcomes.

1. Community Profiling: Mapping health determinants and identifying cultural beliefs or barriers to health-seeking behavior.
2. Feasibility and Acceptability: Assessing how well the community-based integrated healthcare model is accepted by tribal residents and stakeholders.
3. Healthcare Service Utilization: Measuring the community participation in health screenings and formal referral systems.
4. Assessment of the feasibility, acceptability, and potential for wider implementation of the HOR-SATHI Healthy Ageing model in tribal communities.
5. Institutional and Policy Impact: Long-term goals include the institutional adoption of the model by state health departments and providing evidence-based inputs for national geriatric and tribal health policies.

### Outcome measures and assessment timeline

#### Detailed data analysis.

A comprehensive statistical analysis plan for this study employs a multi-layered approach to address both quantitative and qualitative data strands, ensuring that the results are robust and account for the complexities of a tribal setting.

1. Quantitative Analysis Plan

- Quantitative data will be processed and analysed using Jamovi statistical software.
- Descriptive Statistics: Continuous variables, including age, ADL/IADL scores, and WHOQOL-BREF scores, will be summarised using means, medians, and standard deviations.
- Categorical variables, including gender, tribal group, and chronic disease status, will be presented as frequencies and percentages.
- Bivariate Analysis: Chi-square tests will be used to assess associations between categorical variables, such as gender and functional decline.
- Comparison of Groups: Independent t-tests or ANOVA will be used to compare mean functional and quality-of-life scores across relevant groups, such as age categories and nutritional status.
- Correlation Analysis: Pearson’s or Spearman’s correlation coefficients will be used to examine relationships between functional status (ADL/IADL) and quality-of-life measures.

2. Qualitative Analysis Plan

- The qualitative component will provide contextual insights to complement and interpret the quantitative findings through systematic coding and analysis.
  - Thematic Analysis: Transcripts from In-Depth Interviews (IDIs), Focus Group Discussions (FGDs), and Key Informant Interviews (KIIs) will be systematically coded and analysed thematically to identify recurring patterns, cultural beliefs, healthcare experiences, and barriers to healthcare access. Transcripts will be coded and analysed using qualitative data management software such as NVivo, MAXQDA, or ATLAS.ti. Codes will be organized into broader themes addressing healthcare access, perceptions of ageing and functional decline, cultural practices, caregiving, and barriers to modern healthcare. Qualitative findings will initially be analysed independently and subsequently integrated with quantitative findings during interpretation to explain the contextual factors underlying the observed patterns.
- Software: Qualitative data coding and management will be supported using software such as NVivo, MAXQDA, or ATLAS.ti.
- Saturation: Data collection will continue until thematic saturation is achieved, with no emergence of substantially new themes or insights.
- Ensuring Qualitative Research Quality
  - Geographical Coverage: Data collection across five blocks of East Singhbhum district will capture variation in experiences across the study area.
  - Ethical Rigour: Transcripts will be anonymised and assigned unique identification codes, with audio recordings securely managed and accurately transcribed.
  - Community Sensitisation: Village-level sensitisation meetings will be held prior to data collection to facilitate community engagement and encourage open participation.
  - Thematic Saturation: Data collection will continue until no substantially new themes emerge. If new themes arise, additional interviews may be conducted, as permitted by the protocol.
  - Triangulation: Use of IDIs, FGDs, and KIIs and inclusion of diverse stakeholder groups will strengthen the credibility and depth of findings.
  - COREQ- A 32-item checklist designed to improve the transparency and reporting quality of qualitative studies will be employed.

3. Integration of Findings

The quantitative and qualitative data will be analysed independently and integrated at the interpretation stage.

- Quantitative findings: Establish the prevalence and measurable patterns of functional decline, quality of life, and related health conditions.
- Qualitative findings: Provide contextual insights into the cultural, social, and healthcare-related factors underlying these findings.
- Integrated interpretation: The combined findings will directly inform the three-step healthcare model development process, beginning with domain identification and confirmation based on both quantitative evidence and qualitative themes.

4. Managing Confounding Factors

- To minimize potential confounding, multivariable logistic regression models will be used to assess the independent associations with functional decline and quality of life.
- Adjustment for Confounders: Models will adjust for potential confounders, including age, gender, nutritional status, and the presence of chronic conditions.
- Stratification: Where appropriate, analyses will be stratified by gender to examine differences in health needs, occupational risks, and intervention-related outcomes between men and women.

5. Handling Missing Data

- Prevention of Missing Data: Community sensitisation and support from Sahiya/ASHA workers will be used to strengthen community participation and minimise non-response.
- Management of Missing Values: The extent and pattern of missing data will be assessed. Where appropriate, suitable statistical methods, such as multiple imputation, may be considered during the final analysis to minimise potential bias.
- Data Cleaning and Anonymisation: Data will be systematically checked and cleaned before analysis. Unique identification codes will be used to maintain participant confidentiality and facilitate data management.

#### Sources of bias and mitigation strategies.

The study identifies potential sources of bias related to participant selection, communication, measurement, and data management. Measures will be taken to reduce these biases and improve the validity of the findings.

1. Selection Bias

Source: Selection bias may arise if the study participants do not adequately represent the diversity of tribal populations in the study area.Mitigation: A multistage stratified random sampling approach will be used to enhance representativeness across tribal populations, including Scheduled Tribes (STs) and Particularly Vulnerable Tribal Groups (PVTGs). Villages will be selected across five blocks to ensure adequate geographical coverage.

2. Duplication and Participation Bias

Source: Bias may occur among individuals already participating in similar health interventions or research studies.Mitigation: Individuals currently enrolled in similar health-related interventions or research studies will be excluded to minimize duplication and potential influence on study outcomes.

3. Information and Communication Bias

Source: Differences in literacy, language, and cultural context may affect participants’ understanding of study information and interview questions.Mitigation: Village-level sensitization meetings will be conducted with community leaders, Sahiya/ASHA workers, and residents. Information will be provided in an understandable and culturally appropriate manner. For illiterate participants, consent will be documented through a thumb impression in the presence of a witness. Support from Sahiya/ASHA workers will also be sought to facilitate communication and community engagement.

4. Recall and Measurement Bias

Source: Participant recall and reliance on self-reported information may result in inaccurate reporting of health conditions or functional status.Mitigation: The concurrent mixed-methods approach will enable triangulation of findings across quantitative and qualitative data. Standardized and validated instruments, including the Barthel Index, Lawton IADL Scale, and WHOQOL-BREF, will be used to improve the consistency of measurement.

5. Data Management and Researcher Bias

Source: Access to personal identifiers may potentially influence data handling or compromise participant confidentiality.Mitigation: Participant identifiers will be removed or replaced with unique identification codes during data processing and analysis. Data will be securely managed to maintain confidentiality and support objective analysis.

6. Gender Bias

Source: Gender norms and the predominantly female composition of grassroots healthcare workers may limit the engagement of some male tribal community members with health services.

Mitigation: Preferably include male grassroots healthcare workers and tribal volunteers alongside female workers to facilitate gender-sensitive community engagement and ensure equitable participation and non-discriminatory service delivery.

### Data management plan

Table 5: Data Management Plan outlining data ownership, collection, storage, anonymisation, retention and ethical compliance procedures.

Alignment of the Study with the Sustainable Development Goals (SDGs)

SDG 1: No Poverty – By promoting access to preventive and community-based healthcare, the study aims to reduce out-of-pocket health expenditures and the economic vulnerability of ageing individuals. Early detection and management of functional decline and chronic illnesses can significantly lower the health-related financial burden on families, thereby contributing to poverty alleviation.

SDG 2: Zero Hunger – The study addresses nutritional deficiencies and food insecurity among middle-aged and elderly tribal populations through dietary assessments and interventions. By identifying and mitigating malnutrition, the project supports improved nutritional status, food security, and sustainable dietary practices, thereby directly contributing to better health outcomes.

SDG 3: Good Health and Well-being – The primary focus of the study is to enhance the physical, mental, and social well-being of ageing tribal populations. By integrating community-based care models, health education, and preventive screenings, the study fosters healthier ageing trajectories and promotes functional independence, directly contributing to universal health coverage and lifelong well-being.

SDG 10: Reduced Inequalities – Tribal communities often experience significant health disparities and limited access to essential services. This study works to bridge these gaps by implementing inclusive and culturally appropriate healthcare strategies, thereby reducing health inequalities within vulnerable populations and promoting social equity.

SDG 11: Sustainable Cities and Communities – The promotion of age-friendly environments and community-based support systems aligns with the goal of making human settlements inclusive, safe, and sustainable. Through participatory community engagement and local capacity building, the study encourages the development of supportive ecosystems for older adults.

SDG 17: Partnerships for the Goals – The study emphasizes collaboration with local stakeholders, including community health workers, tribal leaders, non-governmental organizations, and government agencies. By fostering multisectoral partnerships and knowledge-sharing platforms, the project strengthens implementation mechanisms and encourages sustainable community ownership of health interventions [11].

### Ethical approval

The study was approved by the Institutional Ethics Committee of Manipal Tata Medical College, Manipal Academy of Higher Education, Jamshedpur, India (IEC Approval No. MTMC/IEC/2025/170). Written informed consent will be obtained from all participants before enrolment.

### Data collection timeline

#### Phase 1.

The study is planned over 36 months following registration. The initial phases will include a literature review, ethical approvals, and questionnaire design and validation. Participant recruitment commenced on 15 May 2026 and will be over by 30^th^ June 2026. Simultaneously, quantitative and qualitative data will be collected from middle-aged and elderly tribal populations in selected blocks of East Singhbhum district. The data collection process is expected to be completed by 15^th^ September 2026, followed by analysis by 15th November 2026, thus providing us with the results of our needs assessment.

#### Phase 2.

**Fig 4:**
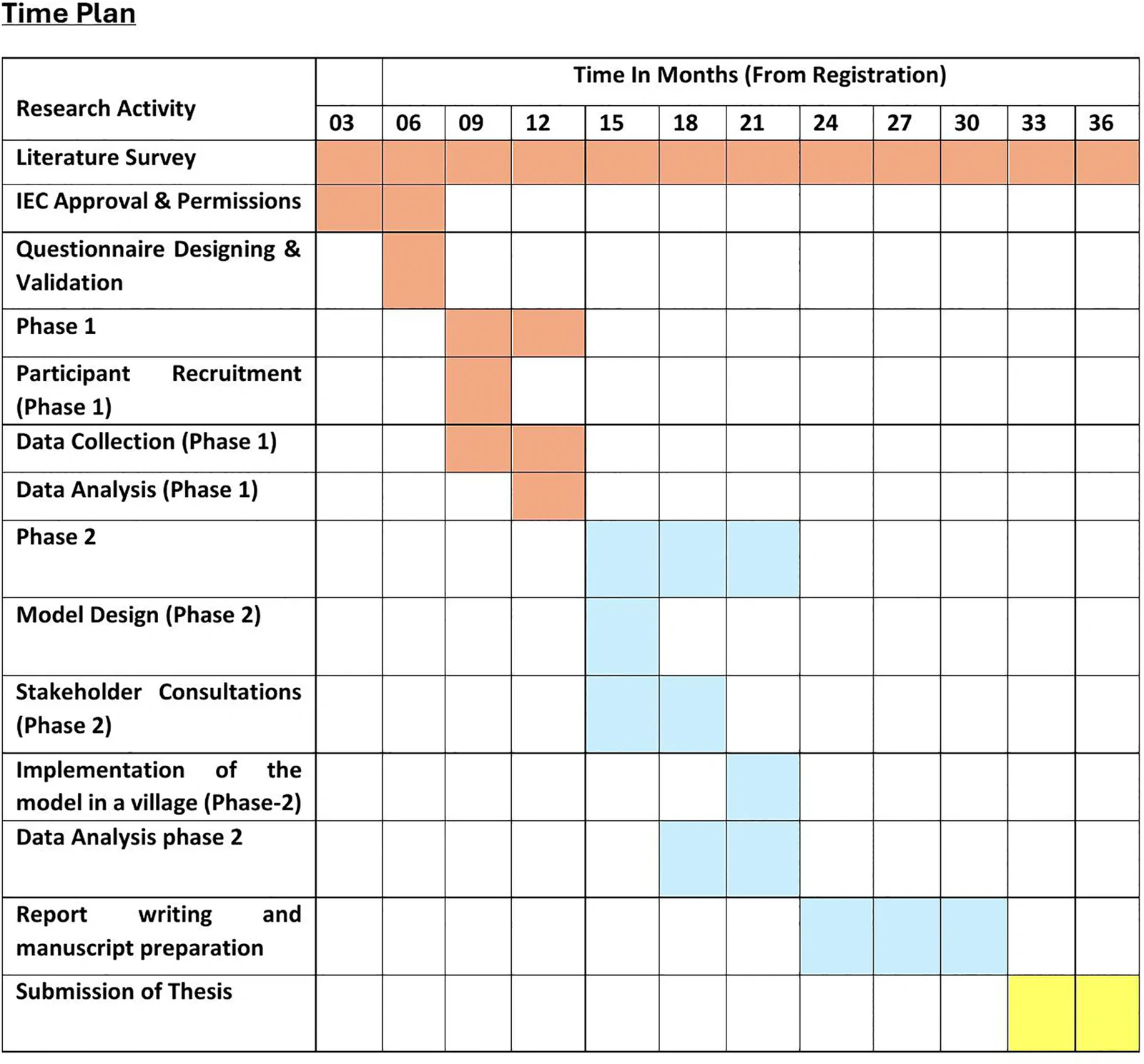
Timeline of research activities across study phases, indicating key milestones from coursework and data collection to model development, implementation and thesis submission.

Following completion and analysis of the needs assessment, Phase 2 of the study will be initiated. Recruitment of stakeholders for Phase 2 is expected to start tentatively on 16 November 2026. Activities during this phase, including development of the community-based integrated healthcare model and stakeholder consultations, will be carried out between November 2026 and May 2027. Subsequently, the model will be implemented in a selected village from June to August 2027. Data analysis related to Phase 2 will be conducted between September and November 2027, followed by report writing, manuscript preparation, and the final thesis. A detailed timeline of the study activities and milestones across both phases of the research is illustrated in Fig 4.

**Time Plan**

## Supporting information

S1 Appendix Questionnaires and data collection instruments. Annexures containing the questionnaires and data collection instruments used for quantitative and qualitative data collection.
(DOCX)
