# Supplementary material for: Strengthening healthy aging through community-based integrated care: A study protocol evaluating functional decline and quality of life among middle-aged and elderly tribals in Jharkhand
Source: PLoS One. 2026 Sep 21;21(9):e0358356. doi: 10.1371/journal.pone.0358356 (PMC13592638; doi:10.1371/journal.pone.0358356)
Supplement: Supplementary file 1 [file pone.0358356.s001.docx]

Sociodemographic Data Collection Form

This form is intended to collect sociodemographic information for a public health survey among tribal and PVTG populations in Jharkhand.

## Identification Details

- Participant ID / Code: ___________
- Date of Interview: ___________
- Village / Hamlet: ___________
- Block: ___________
- District: ___________
- GPS Coordinates (if available): ___________

## Basic Demographic Information

- Name of Respondent: ___________
- Age (in completed years): ___________
- Date of Birth: ___________
- Sex: [ ] Male	[ ] Female	[ ] Other
- Marital Status: [ ] Single	[ ] Married	[ ] Widowed	[ ] Divorced/Separated

## Education and Occupation

- Highest Education Level: [ ] No formal education [ ] Primary [ ] Secondary [ ] Higher Secondary [ ] Graduate or higher
- Occupation: ___________
- Type of Work: [ ] Skilled	[ ] Unskilled	[ ] Agricultural	[ ] Homemaker	[ ] Unemployed	[ ] Retired

## Household and Income Details

- Total Household Members: ___________
- No. of Dependents: ___________
- Annual Household Income (INR): ___________
- Source(s) of Income: ___________

## Caste, Tribe, and Religion

- Caste Category: [ ] ST [ ] SC [ ] OBC [ ] General
- Tribe Name (if ST): ___________
- PVTG Status: [ ] Yes	[ ] No
- If Yes, Specify PVTG Group (e.g., Asur, Birhor): ___________
- Religion: [ ] Hindu [ ] Muslim [ ] Christian [ ] Indigenous/Animist [ ] Other: ___________

## Housing and Living Conditions

- Type of House: [ ] Kutcha [ ] Semi-pucca [ ] Pucca
- Ownership: [ ] Owned [ ] Rented [ ] Shared/Other
- Electricity: [ ] Yes [ ] No
- Drinking Water Source: [ ] Piped [ ] Well [ ] Handpump [ ] River/Stream [ ] Other: ___________
- Sanitation Facility: [ ] Own toilet [ ] Shared [ ] Open defecation
- Cooking Fuel: [ ] LPG [ ] Firewood [ ] Coal [ ] Dung cakes [ ] Other: ___________

## Health Access and Insurance

- Distance to Nearest Health Facility (km): ___________
- Mode of Transport to Health Facility: ___________
- Health Insurance Coverage: [ ] Yes [ ] No
- Type of Insurance (if yes): [ ] PMJAY [ ] ESI [ ] Private [ ] Other: ___________

## Cultural and Social Practices

- Primary Language Spoken at Home: ___________
- Use of Traditional Healers: [ ] Yes [ ] No
- Participation in Government Schemes (BPL, Antyodaya, etc.): [ ] Yes [ ] No
- Any Seasonal Migration in the Last Year: [ ] Yes [ ] No

| **THE** | **Patient Name**: |  |
| --- | --- | --- |
| **BARTHEL** | **Rater Name:** |  |
| **INDEX**  **Activity** | **Date:** | **Score** |
| **FEEDING** |  |  |

0 = unable

5 = needs help cutting, spreading butter, etc., or requires modified diet 10 = independent

## BATHING

0 = dependent

5 = independent (or in shower)

## GROOMING

0 = needs to help with personal care

5 = independent face/hair/teeth/shaving (implements provided)

## DRESSING

0 = dependent

5 = needs help but can do about half unaided

10 = independent (including buttons, zips, laces, etc.)

## BOWELS

0 = incontinent (or needs to be given enemas) 5 = occasional accident

10 = continent

## BLADDER

0 = incontinent, or catheterized and unable to manage alone 5 = occasional accident

10 = continent

## TOILET USE

0 = dependent

5 = needs some help, but can do something alone 10 = independent (on and off, dressing, wiping)

## TRANSFERS (BED TO CHAIR AND BACK)

0 = unable, no sitting balance

5 = major help (one or two people, physical), can sit 10 = minor help (verbal or physical)

15 = independent

## MOBILITY (ON LEVEL SURFACES)

0 = immobile or < 50 yards

5 = wheelchair independent, including corners, > 50 yards

10 = walks with help of one person (verbal or physical) > 50 yards

15 = independent (but may use any aid; for example, stick) > 50 yards

## STAIRS

0 = unable

5 = needs help (verbal, physical, carrying aid) 10 = independent

# TOTAL (0–100):

**The Barthel ADL Index: Guidelines**

1. The index should be used as a record of what a patient does, not as a record of what a patient could do.
2. The main aim is to establish degree of independence from any help, physical or verbal, however minor and for whatever reason.
3. The need for supervision renders the patient not independent.
4. A patient's performance should be established using the best available evidence. Asking the patient, friends/relatives and nurses are the usual sources, but direct observation and common sense are also important. However direct testing is not needed.
5. Usually the patient's performance over the preceding 24-48 hours is important, but occasionally longer periods will be relevant.
6. Middle categories imply that the patient supplies over 50 per cent of the effort.
7. Use of aids to be independent is allowed.

# References

Mahoney FI, Barthel D. “Functional evaluation: the Barthel Index.”

*Maryland State Medical Journal* 1965;14:56-61. Used with permission.

Loewen SC, Anderson BA. “Predictors of stroke outcome using objective measurement scales.”

[***Stroke.* 1990;21:78-81.**](http://www.ncbi.nlm.nih.gov/entrez/query.fcgi?cmd=Retrieve&db=PubMed&list_uids=2300994&dopt=Abstract)

Gresham GE, Phillips TF, Labi ML. “ADL status in stroke: relative merits of three standard indexes.”

[***Arch Phys Med Rehabil.* 1980;61:355-358.**](http://www.ncbi.nlm.nih.gov/entrez/query.fcgi?cmd=Retrieve&db=PubMed&list_uids=7406673&dopt=Abstract)

Collin C, Wade DT, Davies S, Horne V. “The Barthel ADL Index: a reliability study.”

[***Int Disability Study.*1988;10:61-63.**](http://www.ncbi.nlm.nih.gov/entrez/query.fcgi?cmd=Retrieve&db=PubMed&list_uids=3403500&dopt=Abstract)

# Copyright Information

The Maryland State Medical Society holds the copyright for the Barthel Index. It may be used freely for non- commercial purposes with the following citation:

Mahoney FI, Barthel D. “Functional evaluation: the Barthel Index.”

*Maryland State Med Journal* 1965;14:56-61. Used with permission.

Permission is required to modify the Barthel Index or to use it for commercial purposes.

**Patient Name:	Date:	Patient ID #**

| **LAWTON - BRODY**  **INSTRUMENTAL ACTIVITIES OF DAILY LIVING SCALE (I.A.D.L.)** | | | |
| --- | --- | --- | --- |
| **Scoring:** For each category, circle the item description that most closely resembles the client’s highest functional level (either 0 or 1). | | | |
| **A. Ability to Use Telephone** |  | **E. Laundry** |  |
| 1. Operates telephone on own initiative-looks up and dials numbers, etc. 2. Dials a few well-known numbers 3. Answers telephone but does not dial 4. Does not use telephone at all | 1  1  1  0 | 1. Does personal laundry completely 2. Launders small items-rinses stockings, etc. 3. All laundry must be done by others | 1  1  0 |
| **B. Shopping** |  | **F. Mode of Transportation** |  |
| 1. Takes care of all shopping needs | 1 | 1. Travels independently on public transportation or drives own car 2. Arranges own travel via taxi, but does not otherwise use public transportation 3. Travels on public transportation when accompanied by another 4. Travel limited to taxi or automobile with assistance of another 5. Does not travel at all | 1 |
| independently |  |  |  |
| 2. Shops independently for small purchases | 0 |  | 1 |
| 3. Needs to be accompanied on any shopping | 0 |  |  |
| trip |  |  | 1 |
| 4. Completely unable to shop | 0 |  |  |
|  |  |  | 0 |
|  |  |  | 0 |
| **C. Food Preparation** |  | **G. Responsibility for Own Medications** |  |
| 1. Plans, prepares and serves adequate meals independently 2. Prepares adequate meals if supplied with ingredients 3. Heats, serves and prepares meals, or prepares meals, or prepares meals but does not maintain adequate diet 4. Needs to have meals prepared and served | 1 | 1. Is responsible for taking medication in correct dosages at correct time 2. Takes responsibility if medication is prepared in advance in separate dosage 3. Is not capable of dispensing own medication | 1 |
|  | 0 |  | 0 |
|  | 0 |  | 0 |
|  | 0 |  |  |
| **D. Housekeeping** |  | **H. Ability to Handle Finances** |  |
| 1. Maintains house alone or with occasional assistance (e.g. "heavy work domestic help") 2. Performs light daily tasks such as dish washing, bed making 3. Performs light daily tasks but cannot maintain acceptable level of cleanliness 4. Needs help with all home maintenance tasks 5. Does not participate in any housekeeping tasks | 1 | 1. Manages financial matters independently (budgets, writes checks, pays rent, bills, goes to bank), collects and keeps track of income 2. Manages day-to-day purchases, but needs help with banking, major purchases, etc. 3. Incapable of handling money | 1 |
|  | 1 |  |  |
|  |  |  | 1 |
|  | 1 |  |  |
|  |  |  | 0 |
|  | 1 |  |  |
|  | 0 |  |  |
| **Score** |  | **Score** |  |
| **Total score**  A summary score ranges from 0 (low function, dependent) to 8 (high function, independent) for women and 0 through 5 for men to avoid potential gender bias. | | |  |

WHOQOL-BREF

June 1997

U.S. Version

University of Washington Seattle, Washington United States of America

Emblem...Soul Catcher: a Northwest Coast Indian symbol of physical and mental well-being. Artist: Marvin Oliver

**WHOQOL-BREF**

**About You**

Before you begin we would like to ask you to answer a few general questions about yourself by circling the correct answer or by filling in the space provided.

| 1. What is your gender | Male | Female |
| --- | --- | --- |

| 2. What is your date of birth? | / | / |
| --- | --- | --- |
| Day | Month | Year |

| 3. What is the highest education you  received? | None at all |
| --- | --- |
|  | Elementary School |
|  | High School |
|  | College |

| 4. What is your marital status? | Single | Separated |
| --- | --- | --- |
|  | Married | Divorced |
|  | Living as Married | Widowed |

| 5. Are you currently ill? | Yes | No |
| --- | --- | --- |
| 6. If something is wrong with your health, what do you  think it is? |  | illness/problem |

Instructions

This questionnaire asks how you feel about your quality of life, health, or other areas of your life. Please answer all the questions. If you are unsure about which response to give to a question, please choose the one that appears most appropriate. This can often be your first response.

Please keep in mind your standards, hopes, pleasures and concerns. We ask that you think about your life in the last two weeks. For example, thinking about the last two weeks, a question might ask:

| *(Please circle the number)* | | | | | |
| --- | --- | --- | --- | --- | --- |
| *For office use* | **Not at all** | **A little** | **Moderately** | **Mostly** | **Completely** |
| Do you get the kind of support from others that you need? | **1** | **2** | **3** | **4** | **5** |

You should circle the number that best fits how much support you got from others over the last two weeks. So you would circle the number 4 if you got a great deal of support from others. o

| *(Please circle the number)* | | | | | |
| --- | --- | --- | --- | --- | --- |
| *For office use* | **Not at all** | **A little** | **Moderately** | **Mostly** | **Completely** |
| Do you get the kind of support from others that you need? | **1** | **2** | **3** | **4**  | **5** |

You would circle number 1 if you did not get any of the support that you needed from others in the last two weeks. o

| *(Please circle the number)* | | | | | |
| --- | --- | --- | --- | --- | --- |
| *For office use* | **Not at all** | **A little** | **Moderately** | **Mostly** | **Completely** |
| Do you get the kind of support from others that you need? | **1**  | **2** | **3** | **4** | **5** |

Please read each question, assess your feelings, and circle the number on the scale that gives the best answer for you for each question.

| *(Please circle the number)* | | | | | | |
| --- | --- | --- | --- | --- | --- | --- |
| *For office use* | **Very poor** | | **Poor** | **Neither poor nor good** | **Good** | **Very Good** |
| G1 / G1.1 | 1. How would you rate your quality of life? | **1** | **2** | **3** | **4** | **5** |

| *(Please circle the number)* | | | | | | |
| --- | --- | --- | --- | --- | --- | --- |
| *For office use* |  | **Very dissatisfied** | **Dissatisfied** | **Neither**  **satisfied nor dissatisfied** | **Satisfied** | **Very satisfied** |
| G4 / G2.3 | 2. How satisfied are you with your health? | **1** | **2** | **3** | **4** | **5** |

The following questions ask about **how much** you have experienced certain things in the last two weeks.

| *(Please circle the number)* | | | | | | |
| --- | --- | --- | --- | --- | --- | --- |
| *For office use* |  | **Not at all** | **A little** | **A moderate amount** | **Very much** | **An extreme amount** |
| F1.4 / F1.2.5 | 3. To what extent do you feel that physical pain prevents you from doing what you need to do? | **1** | **2** | **3** | **4** | **5** |
| F11.3 / F13.1.4 | 4. How much do you need any medical treatment to function in your daily life? | **1** | **2** | **3** | **4** | **5** |
| F4.1 / F6.1.2 | 5. How much do you enjoy life? | **1** | **2** | **3** | **4** | **5** |

| *(Please circle the number)* | | | | | | |
| --- | --- | --- | --- | --- | --- | --- |
| *For office use* | **Not at all** | | **A little** | **A moderate amount** | **Very much** | **An extreme amount** |
| F24.2 / F29.1.3 | 6. To what extent do you feel your life to be meaningful? | **1** | **2** | **3** | **4** | **5** |

| *(Please circle the number)* | | | | | | |
| --- | --- | --- | --- | --- | --- | --- |
| *For office use* |  | **Not at all** | **Slightly** | **A Moderate amount** | **Very much** | **Extremely** |
| F5.2 / F7.1.6 | 7. How well are you able to concentrate? | **1** | **2** | **3** | **4** | **5** |
| F16.1 / F20.1.2 | 8. How safe do you feel in your daily life? | **1** | **2** | **3** | **4** | **5** |
| F22.1 / F27.1.2 | 9. How healthy is your physical environment? | **1** | **2** | **3** | **4** | **5** |

The following questions ask about **how completely** you experience or were able to do certain things in the last two weeks.

| *(Please circle the number)* | | | | | | |
| --- | --- | --- | --- | --- | --- | --- |
| *For office use* |  | **Not at all** | **A little** | **Moderately** | **Mostly** | **Completely** |
| F2.1 / F2.1.1 | 10. Do you have enough energy for everyday life? | **1** | **2** | **3** | **4** | **5** |
| F7.1 / F9.1.2 | 11. Are you able to accept your bodily appearance? | **1** | **2** | **3** | **4** | **5** |
| F18.1 / F23.1.1 | 12. Have you enough money to meet your needs? | **1** | **2** | **3** | **4** | **5** |

| *(Please circle the number)* | | | | | | |
| --- | --- | --- | --- | --- | --- | --- |
| *For office use* |  | **Not at all** | **A little** | **Moderately** | **Mostly** | **Completely** |
| F20.1 / F25.1.1 | 13. How available to you is the information that you need in your day-to-day life? | **1** | **2** | **3** | **4** | **5** |
| F21.1 / F26.1.2 | 14. To what extent do you have the opportunity for leisure activities? | **1** | **2** | **3** | **4** | **5** |

| *(Please circle the number)* | | | | | | |
| --- | --- | --- | --- | --- | --- | --- |
| *For office use* |  | **Very poor** | **Poor** | **Neither poor nor well** | **Well** | **Very well** |
| F9.1 / F11.1.1 | 15. How well are you able to get around? | **1** | **2** | **3** | **4** | **5** |

The following questions ask you to say how **good** or **satisfied** you have felt about various aspects of your life over the last two weeks.

| *(Please circle the number)* | | | | | | |
| --- | --- | --- | --- | --- | --- | --- |
| *For office use* | **Very dissatisfied** | | **Dissatisfied** | **Neither satisfied nor**  **dissatisfied** | **Satisfied** | **Very satisfied** |
| F3.3 / F4.2.2 | 16. How satisfied are you with your sleep? | **1** | **2** | **3** | **4** | **5** |
| F10.3 / F12.2.3 | 17. How satisfied are you with your ability to perform your daily living activities? | **1** | **2** | **3** | **4** | **5** |
| F12.4 / F16.2.1 | 18. How satisfied are you with your capacity for work? | **1** | **2** | **3** | **4** | **5** |

| *(Please circle the number)* | | | | | | |
| --- | --- | --- | --- | --- | --- | --- |
| *For office use* | **Very dissatisfied** | | **Dissatisfied** | **Neither satisfied nor**  **dissatisfied** | **Satisfied** | **Very satisfied** |
| F6.4 / F8.2.2 | 19. How satisfied are you with yourself? | **1** | **2** | **3** | **4** | **5** |
| F13.3 / F17.2.3 | 20. How satisfied are you with your personal relationships? | **1** | **2** | **3** | **4** | **5** |
| F15.3 / F3.2.1 | 21. How satisfied are you with your sex life? | **1** | **2** | **3** | **4** | **5** |
| F14.4 / F18.2.5 | 22. How satisfied are you with the support you get from your friends? | **1** | **2** | **3** | **4** | **5** |
| F17.3 / F21.2.2 | 23. How satisfied are you with the conditions of your living place? | **1** | **2** | **3** | **4** | **5** |
| F19.3 / F24.2.1 | 24. How satisfied are you with your access to health services? | **1** | **2** | **3** | **4** | **5** |
| F.23.3 / F28.2.2 | 25. How satisfied are you with your mode of transportation? | **1** | **2** | **3** | **4** | **5** |

The follow question refers to **how often** you have felt or experienced certain things in the last two weeks.

| *(Please circle the number)* | | | | | | |
| --- | --- | --- | --- | --- | --- | --- |
| *For office use* |  | **Never** | **Seldom** | **Quite often** | **Very often** | **Always** |
| F8.1 / F10.1.2 | 26. How often do you have negative feelings, such as blue mood, despair, anxiety, depression? | **1** | **2** | **3** | **4** | **5** |

| Did someone help you to fill out this  form? *(Please circle Yes or No)* | Yes | No |
| --- | --- | --- |

How long did it take to fill out this form?

**Thank you for your help**

WHOQOL-BREF, Questionnaire, June 1997, Updated 1/10/2014	8

***Domain Scores***

| **Domains** | **WHOQOL-100 Facets** | Raw domain score | **Raw score range** |
| --- | --- | --- | --- |
| Domain 1: Physical | Facet 1 + Facet 2 + Facet 3 | 12 - 60 | 48 |
| Domain 2: Psychological | Facet 4 + Facet 5 + Facet 6 +  Facet 7 + Facet 8 | 20 – 100 | 80 |
| Domain 3: Level of Independence | Facet 9 + Facet 10 + Facet 11 +  Facet 12 | 16 – 80 | 64 |
| Domain 4: Social relationships | Facet 13 + Facet 14 + Facet 15 | 12 – 60 | 48 |
| Domain 5: Environment | Facet 16 + Facet 17 + Facet 18 +  Facet 19 + Facet 20 + Facet 21+  Facet 22 + Facet 23 | 32 – 160 | 128 |
| Domain 6: Spirituality / Religion / Personal beliefs | Facet 24 | 4 – 20 | 16 |

***Transformation of Scale Scores***

The next step involves transforming each raw scale score to a 0-100 scale using the formula shown below:

Transformed Scale = ⎡(Actual raw score - lowest possible raw score) ⎤ ×100

⎢	Possible raw score range	⎥

⎣

⎦

where “Actual raw score” is the values achieved through summation, “lowest possible raw score” is the lowest possible value that could occur through summation (this value would be 4 for all facets), and “Possible raw score range” is the difference between the maximum possible raw score and the lowest possible raw score (this value would be 16 for all facets: 20 minus 4).

This transformation converts the lowest and highest possible scores to zero and 100, respectively. Scores between these values represent the percentage of the total possible score achieved. The WHOQOL-100 scores from other Centers may not be transformed to the 0-100 scale. The U.S.WHOQOL instruments and scoring programs have used this transformation to provide comparative data for interpretation.

*Example: A Facet 1 “Pain and discomfort” raw score of 15 would be transformed as follows:*

Transformed Scale = ⎡(15 - 4) ⎤ ×100 = 68.75

⎢⎣	16	⎥⎦

I:\SEAQOL Instrument Coordination\Instruments\WHOQOL-BREF\WHOQOL Manual.Doc	32

**WHOQOL-BREF Scoring**

The WHOQOL-Bref, still in field trials, is a subset of 26 items taken from the WHOQOL-100. The same steps for the scoring WHOQOL-100 should be followed to achieve scores for the Bref. Although scoring the Bref is identical to scoring the WHOQOL-100, there are some differences that need to be addressed:

- The WHOQOL-Bref does not have facet scores
- Mean substitutions are recommended for Domain 1 *Physical Health* and Domain 4

*Environment* if no more than one item is coded missing

- Only three items need to be reversed before scoring

The WHOQOL-Bref (Field Trial Version) produces a profile with four domain scores and two individually scored items about an individual’s overall perception of quality of life and health. The four domain scores are scaled in a positive direction with higher scores indicating a higher quality of life. Three items of the Bref must be reversed before scoring. They can be seen in Table 9, indicated by the “- (reverse)” denotation in the *Direction of scaling* column.

**TABLE 9. Scoring Domains of the WHOQOL-BREF**

| **Domains and questions 236/BREF** | | **Direction of scaling** | **Raw domain score** | **Raw item score** |
| --- | --- | --- | --- | --- |
| **Overall Quality of Life and General Health** | |  | ....(2-10) |  |
| G1.1/B1 | How would you rate your quality of life? | + |  | ....(1-5) |
| G2.3/B2 | How satisfied are you with your health? | + |  | ....(1-5) |
| **Domain 1** | **Physical Health** |  | ....(7-35) |  |
| F1.2.5/B3 | To what extent do you feel that physical pain prevents you from | -(reverse) |  | ....(1-5) |
|  | doing what you need to do? |  |  |  |
| F13.1.4/B4 | How much do you need any medical treatment to function in | -(reverse) |  | ....(1-5) |
|  | your daily life? |  |  |  |
| F2.1.1/B10 | Do you have enough energy for everyday life? | + |  | ....(1-5) |
| F11.1.1/B15 | How well are you able to get around? | + |  | ....(1-5) |
| F4.1.1/B16 | How satisfied are you with your sleep | + |  | ....(1-5) |
| F12.2.3/B17 | How satisfied are you with your ability to perform your daily | + |  | ....(1-5) |
|  | living activities? |  |  |  |
| F16.2.1/B18 | How satisfied are you with your capacity for work? | + |  | ....(1-5) |
| **Domain 2** | **Psychological** |  | ....(6-30) |  |
| F6.1.2/B5 | How much do you enjoy life? | + |  | ....(1-5) |
| F29.1.3/B6 | To what extent do you feel your life to be meaningful? | + |  | ....(1-5) |
| F7.1.6/B7 | How well are you able to concentrate? | + |  | ....(1-5) |
| F9.1.2/B11 | Are you able to accept your bodily appearance? | + |  | ....(1-5) |
| F8.2.1/B19 | How satisfied are you with yourself? | + |  | ....(1-5) |
| F10.1.2/B26 | How often do you have negative feelings such as blue mood, | - (reverse) |  | ....(1-5) |
|  | despair, anxiety, depression? |  |  |  |
| **Domain 3** | **Social relationships** |  | ....(3-15) |  |
| F17.1.3/B20 | How satisfied are you with your personal relationships? | + |  | ....(1-5) |
| F3.2.1/B21 | How satisfied are you with your sex life? | + |  | ....(1-5) |
| F18.2.5/B22 | How satisfied are with the support you get from your friends? | + |  | ....(1-5) |

I:\SEAQOL Instrument Coordination\Instruments\WHOQOL-BREF\WHOQOL Manual.Doc

35

| **Domains and questions 236/BREF** | **Direction of scaling** | **Raw domain score** | **Raw item score** |
| --- | --- | --- | --- |
| **Domain 4 Environment** |  | ....(8-40) |  |
| F20.1.2/B8 How safe do you feel in your daily life? | + |  | ....(1-5) |
| F27.1.2/B9 How healthy is your physical environment? | + |  | ....(1-5) |
| F23.1.1/B12 Have you enough money to meet your needs? | + |  | ....(1-5) |
| F25.1.1/B13 How available to you is the information that you need in your daily-to-day life? | + |  | ....(1-5) |
| F26.1.2/B14 To what extent do you have the opportunity for leisure activities? | + |  | ....(1-5) |
| F21.2.2/B23 How satisfied are you with the condition of your living place? | + |  | ....(1-5) |
| F24.2.1/B24 How satisfied are you with your access to health services? | + |  | ....(1-5) |
| F28.2.2/B25 How satisfied are you with your transport? | + |  | ....(1-5) |

If no more than one item from the *Physical Health* or *Environment* domains has been coded as missing, we recommend that a domain score be calculated by substituting a person- specific average across the completed items in the same scale. For example, if a respondent does not have a value for item B16 *How satisfied are you with your sleep?* in the Physical Health domain, but has answered all of the other items in that domain, then the value for item B16 would be the average of the remaining 6 items. If two or more items are coded missing in these two domains, the domain score should not be calculated, likewise if any items are coded missing in the *Psychological* and *Social Relationships* domains, a domain score for that respondent would not be calculated.

After item recoding and handling of missing data, a raw score is computed by a simple algebraic sum of each item in each of the four domains. Once complete, check the frequencies of each domain to be sure that the scores are within the correct range indicated in Table 9 *Raw domain score* column. The next step is to transform each raw scale score using the formula on page 32. The possible raw score ranges for each domain are as follows: *Physical Health*=28, *Psychological*=24, *Social Relationships*=12, and *Environment*=32.

***Scoring Exercise and Test Dataset for the WHOQOL-BREF Instrument***

The purpose of this scoring exercise is to help WHOQOL-Bref users to evaluate results from each step in the process of calculating the Domain summary scores of the instrument. This exercise was created for SPSS users, but with minor modifications, can be adapted for other computer programs or can be useful for those scoring the survey manually.

A test dataset and SPSS code for scoring the WHOQOL-Bref a computer disk in this packet. The test dataset, which is called “**WQ_BREF.TXT**” on the disk, contains data from 64 administrations of the WHOQOL-BREF. The data can be seen in *Appendix F*. The enclosed diskette also provides the user with the SPSS syntax used to:

- import raw data into SPSS format [**WQ_B_DL.SPS**]
- derive the WHOQOL-BREF domain summaries [**WQ_BREF.SPS**]

I:\SEAQOL Instrument Coordination\Instruments\WHOQOL-BREF\WHOQOL Manual.Doc	36

The SPSS code (called “**WQ_BREF.SPS**”) on the disk begins by labeling all items and checking for out-or-range values. It then recodes the 3 negatively stated items so that a higher score indicates better health. The 4 domains are then scored, labeled, and transformed to a 0 to 100 scale used to interpret and compare to other validated instrument tools such as the WHOQOL-100. A copy of the SPSS syntax is reproduced in Appendix F.

Table 10 presents statistics for the transformed domains for the WHOQOL-Bref. After scoring the test dataset, the means, standard deviations, and minimum and maximum observed values should agree with those presented in Table 10

**TABLE 10. Test Dataset Descriptive Statistics: WHOQOL-BREF**

**Descriptive Statistics**

Std.

(TRANSFORMED) (TRANSFORMED) (TRANSFORMED) (TRANSFORMED)

| N | Minimum | Maximum | Mean | Deviation |
| --- | --- | --- | --- | --- |
| Physical _64_ | 32.14 | 92.86 | 66.7969 | 14.5480 |
| Psychological _64_ | 37.50 | 95.83 | 73.5026 | 13.7165 |
| Social Relations _64_ | 25.00 | 100.00 | 73.1771 | 17.0891 |
| Environment _64_ | 28.13 | 100.00 | 72.8027 | 14.1592 |
| Valid N (listwise) 64 |  |  |  |  |

I:\SEAQOL Instrument Coordination\Instruments\WHOQOL-BREF\WHOQOL Manual.Doc

37

✁
